# Trabecular Bone Adaptation to Low-Magnitude High-Frequency Loading in Microgravity

**DOI:** 10.1371/journal.pone.0093527

**Published:** 2014-05-02

**Authors:** Antonia Torcasio, Katharina Jähn, Maarten Van Guyse, Pieter Spaepen, Andrea E. Tami, Jos Vander Sloten, Martin J. Stoddart, G. Harry van Lenthe

**Affiliations:** 1 Biomechanics Section, KU Leuven, Leuven, Belgium; 2 AO Research Institute, Davos, Switzerland; 3 Institute for Biomechanics, ETH Zurich, Zurich, Switzerland; University Hospital of the Albert-Ludwigs-University Freiburg, Germany

## Abstract

Exposure to microgravity causes loss of lower body bone mass in some astronauts. Low-magnitude high-frequency loading can stimulate bone formation on earth. Here we hypothesized that low-magnitude high-frequency loading will also stimulate bone formation under microgravity conditions. Two groups of six bovine cancellous bone explants were cultured at microgravity on a Russian Foton-M3 spacecraft and were either loaded dynamically using a sinusoidal curve or experienced only a static load. Comparable reference groups were investigated at normal gravity. Bone structure was assessed by histology, and mechanical competence was quantified using μCT and FE modelling; bone remodelling was assessed by fluorescent labelling and secreted bone turnover markers. Statistical analyses on morphometric parameters and apparent stiffness did not reveal significant differences between the treatment groups. The release of bone formation marker from the groups cultured at normal gravity increased significantly from the first to the second week of the experiment by 90.4% and 82.5% in response to static and dynamic loading, respectively. Bone resorption markers decreased significantly for the groups cultured at microgravity by 7.5% and 8.0% in response to static and dynamic loading, respectively. We found low strain magnitudes to drive bone turnover when applied at high frequency, and this to be valid at normal as well as at microgravity. In conclusion, we found the effect of mechanical loading on trabecular bone to be regulated mainly by an increase of bone formation at normal gravity and by a decrease in bone resorption at microgravity. Additional studies with extended experimental time and increased samples number appear necessary for a further understanding of the anabolic potential of dynamic loading on bone quality and mechanical competence.

## Introduction

One of the effects resulting from the exposure to microgravity is the loss of bone mass in the lower limbs and spine in some astronauts [1], [2]. Data from spaceflights on the Russian space station Mir and the International Space Station ISS have demonstrated the impact of weightlessness on bone volume. During a space mission at the Mir, areal bone mineral density (aBMD) of some astronauts was reduced by a monthly rate of 0.3% from the total skeleton, with 97% of that loss occurring in the pelvis and legs [3]. These measurements varied largely between astronauts; some individuals incurred losses equivalent to one-half the bone mineral they would lose in a lifetime of normal aging, while others experienced only a small BMD reduction.

In the spine and hip, relevant areas of frequently seen osteoporotic bone fractures in the elderly, a decrease in aBMD of 0.9% and 1.5% per month respectively was found in ISS crew members [4]. Computed Tomography (CT) measurements in combination with DXA (dual energy X-ray absorptiometry) revealed that bone is lost in cancellous as well as in cortical bone volumes. These effects on bones might not have immediate implications for the astronauts, but could lead to increased fracture risk because of an associated loss in bone strength as well as to an early onset of age-related osteoporosis in the more advanced age.

As a potential countermeasure to prevent bone loss, mechanical stimulation has attracted much attention in recent years. It is well known that bone is capable of adapting its mass and structure in response to mechanical loading. The nature of the mechanical stimulus for bone adaptation has been debated for over 100 years [5]. Researchers initially showed that the adaptive response of bone to loading is influenced by the mechanical strain magnitude and demonstrated that bone formation is initiated when a certain strain threshold is surpassed. An important contribution was made in 1971 by Hert and co-workers when they showed that dynamic, but not static, strains increased bone formation in rabbits [6]. Dynamic strains thus appeared to be the primary stimulus of bone adaptation. O'Connor et al [7] emphasized the influence of the rate of strain, more specifically that high frequency components of the loading cycle were important for maximal bone response. With incremental increases in strain, bone responds with further increases in formation activity [8]. By decomposing the mechanical stimulus into constituent components, researchers have subsequently identified several mechanical parameters that influence the anabolic response to loading. Among these are strain rate, strain distribution, local strain gradients [9], number of cycles [10], and resting periods [11], [12]. Strain rate can be further decomposed into strain magnitude and loading frequency. Low-amplitude high-frequency loading in bone has been shown to occur more often in normal daily activities in vivo [13] and has been investigated as a loading regime to promote adaptive bone formation. Specifically, it has been demonstrated that low-amplitude high-frequency stimuli (500 microstrains at 30 Hz) were sufficient to stimulate new bone formation in experimental animals, whereas high-amplitude low-frequency (3000 microstrains at 1 Hz) was insufficient [14]. The tissue's sensitivity to low-amplitude high-frequency loading suggests an interesting potential pathway for therapeutic intervention, as the adaptive capacity of bone in response to such loading could be considered as a potential countermeasure to prevent bone loss under weightlessness conditions.

For the present study we hypothesized that low-amplitude (apparent strain <500 microstrains) high-frequency (30 Hz) mechanical loading would influence the bone remodelling processes in microgravity conditions also. In addition, we addressed the question whether the amount of bone adaptation is related to strain magnitude. In order to test these hypotheses, an explant culture system that provides mechanical loading, in addition to managing nutrition and waste products [15], [16] was used for the first time in space.

## Materials and Methods

Fully automated microgravity experiments were conducted on board of the unmanned Russian Foton-M3 spacecraft, launched from Kazakhstan on September 14, 2007. When Foton-M3 was in orbit, a reference experiment at 1 g was conducted on ground. The mission duration was 12 days. After the experiments, the bone samples and perfusion media were collected. First, bone turnover markers (C-terminal propeptide of type I collagen and N-terminal telopeptide of type I collagen) were quantified to analyse whether trabecular bone adapts to dynamic loading at normal and microgravity. Second, histological evaluation of the bone was performed to investigate the morphology of the bone tissue and determine the presence of osteoblasts and osteoclasts. Third, we evaluated bone quality, in terms of its micro-architecture and mechanical stiffness, in response to mechanical loading at normal and microgravity. Finally, we investigated whether the bone adaptation processes may be linked to strain levels within the trabeculae.

### The FreqBone experiment

Four different groups each containing six bovine cancellous bone explants were taken from the sternum of a 1-year old cow. Given the restrictions related to performing an experiment in space, only a limited number of specimens per group was investigated. All samples were obtained from one animal in order to avoid any inter-individual variations. The sternum was ordered from a local butcher (Van den Berg, Voorhout, the Netherlands). Animal euthanasia was performed in order to guarantee the quality of the tissue. Procedures were checked in person by a veterinary of the Netherlands Food and Consumer Product Safety Authority. The sternum was considered a normal product of slaughter; hence, no ethics approval was required. Two groups were cultured at microgravity and were either loaded dynamically using a sinusoid curve (30 N peak to peak amplitude and 30 Hz frequency), or experienced only a static load of 30 N. Two reference groups were cultured at normal gravity and experienced either static or dynamic load identical to the groups at microgravity.

The preparation of the cylindrical bone explants (diameter  = 10 mm, height  = 5 mm), as well as the culture medium (Dulbecco's Modified Eagle Medium DMEM +10% Fetal Calf Serum FCS) was performed at the European Space Agency (ESA) Research and Technology Centre in Noordwijk (Netherlands).

**Figure 1:**
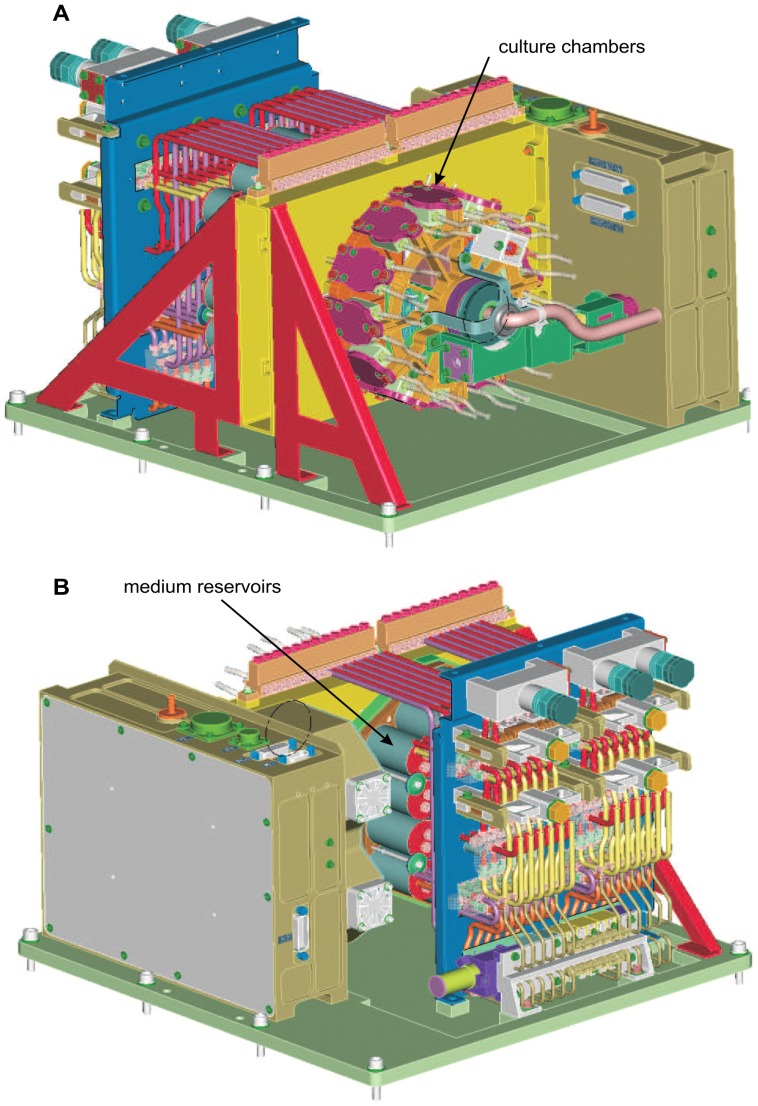
Schematic illustration of the FreqBone bioreactor and culture system. A) The front side shows the culture chambers that are arranged in a circle with a rotating loading stack in the centre; B) The medium reservoirs and the pump system are visible on the back side.

The bovine sternum was cleaned of remaining soft tissues. With the use of an ‘Exakt 300’ band saw (Exakt Apparatebau GmbH & Co. KG) the bone was cut into 7 mm-thick sections. Cancellous bone cores were drilled out from these sections using an EcoMac 212 bench drill containing a Synthes drill bit (Ref: 387.661, Synthes, Bettlach, Switzerland), resulting in 10 mm diameter cancellous bone cores. Cores were then grounded parallel to 5 mm height. During all cutting and drilling procedures, the bone explants were irrigated with sterile pre-cooled (4°C) saline (0.9% sodium chloride solution) to reduce the formation of bone debris and heat-induced cell death. Adherent bone debris was removed by washing each explant three times in 10 ml HBSS (Hank's Buffered Salt Solution) for 30 min each at 4°C. Each cancellous bone explant was then inserted inside a culture chamber (Mathys, CH) under sterile conditions. The FreqBone culture and bioreactor system containing the bone samples was brought to Baikonur (Kazakhstan). The normal gravity experiment with the reference samples was performed in Leuven (Belgium). All bone explants and culture media were kept at 10°C for 4.5 days prior to the start of the experiment. The experiment was started on board of the spacecraft immediately after launch by raising the temperature of the explants from 10°C to 37°C; the temperature of the explants was maintained at 37°C for the duration of the flight. The explants were subjected to mechanical loading for 12 days continuously. The FreqBone bioreactor and culture system had been developed on the basis of the technology previously used in the ‘Zetos’ system [17]. Extensive cell viability investigations on the Zetos system have been reported [17], [18]. In short, the culture chambers containing the bone explants were perfused with a pump system connected to the culture medium reservoirs. For mechanical stimulation, the chambers were in circular arrangement with a circulating loading stack in the centre [19] (Figure 1).

At two different time points of the FreqBone experiment, bone explants were perfused with fluorescent labels in order to detect small changes in bone formation. Specifically, calcein green was applied prior to launch and alizarin complexon was given after the mission. The labelling was performed at 10°C to avoid high metabolic activity of the explants and, therefore, uptake of the dye at active surfaces [20]. Each label was applied for 24 h.

The culture medium was exchanged once after 6 days, hence, two medium samples per explant (day 6 and day 12) were used for analyses. Media were kept at 10°C and were heated to 37°C just prior to bone explant contact. Each explant was cultured with 20 ml medium for 6 days prior to exchange. When the 12-day spaceflight was over, bone explants were stored for 2 days at 10°C prior to fixation in 70% ethanol. Culture media collected at day 6 and day 12 were frozen and stored for further analysis. All reference specimens were treated the same as experimental group specimens with the exception that the reference specimens were not flown to Kazakhstan.

### Culture medium analyses

The soluble products in the culture medium that arise from matrix breakdown and formation were quantified. All medium investigations were performed on one day to avoid repeated freezing and thawing of the samples and to keep analyses consistency. Analyses of the medium samples involved the detection of the C-terminal propeptide of type I collagen (ProCI) as a bone formation marker, and of N-terminal telopeptide of type I collagen (NTx) as indicator of bone resorption.

The release of ProCI was quantified using the “Procollagen Type I C-Peptide Enzyme Immunoassay Kit” (Takara Bio Inc., Shiga, Japan) according to manufacturer's instructions. Briefly, 20 µl sample or standard (with a range from 0–640 ng ProCI/ml were used in this assay per 96-well. The absorbance of the final product was measured at 450 nm using a “HTS 7000 Bio Assay Reader” (Perkin Elmer Perkin-Elmer, Shelton, CT).

The osteoclast-dependent release of NTx was quantified using the “OSTERMARK® NTx-Serum” (Ostex International Inc., Seattle, WA) according to manufacturer's instructions. The culture medium samples were diluted 1∶1 in “specimen diluent”. Samples, “assay calibrators”, and reference samples were used at 100 µl per well in 96-well plates. The optical density of the colour reaction was determined at 450 nm using a “HTS 7000 Bio Assay Reader” (Perkin Elmer Perkin-Elmer, Shelton, CT).

### Histological Analyses

Bone explants were dehydrated in a gradient of ethanol and embedded in Technovit (‘Technovit 9100 New’ kit, Kulzer GmbH) according to manufacturer instructions. Thick sections were prepared from embedded tissue blocks using a Leitz 1600 saw microtome. Grinding and polishing of the sections was performed until a final section thickness of 100 µm was reached. Visualisation of calcein and alizarin fluorescent labelling was performed using an ‘Axioplan 2’ microscope (Zeiss). The analysis of bone formation rate was not possible, due to a lack of a distinct double label. Only single bands of either fluorochrome or a yellow band created by the overlapping of both fluorochrome bands could be detected. Nevertheless, labelling fluorescence indicated the presence of free calcium binding sites either created by osteoblasts or osteoclasts activity. To evaluate cell activity of cells inside the bone explants label penetration into the bone explants was measured. Therefore, fluorescent micrographs from the whole area of the centre section from each explant were stitched together to represent this section of each explant. The maximal label penetration was measured both from the upper and lower surfaces, as well as from the circumference edge. To investigate the morphological state of the cultured bone explants, the same sections used for the visualisation of the fluorescent double labelling were stained with Giemsa and eosin.

### Micro-computed tomography (μCT)

The ethanol-fixed bone explants were imaged using a μCT-40 apparatus (Scanco Medical, Brüttisellen, Switzerland) with a nominal resolution of 10 µm. The X-ray tube was operated at 70 kV and 114 µA. Three-fold oversampling was used with an integration time of 300 ms.

The μCT data of each sample were filtered using a constrained three-dimensional Gaussian filter (σ = 1.2, support  = 2), to partially suppress the noise in the volumes. Bone tissue was separated from marrow using a global threshold (22.4% of maximal gray value). The edge regions containing bone debris derived from sample preparation were excluded from the segmented data resulting in a cylindrical volume of interest with 8 mm diameter and 4 mm height. Image processing was performed using IPL (Scanco Medical, Brüttisellen, Switzerland).

### 3D morphometry

For each model, standard morphometric indices were assessed ([21]). Bone surface area (BS), bone volume (BV) and total volume (TV) were calculated using the software program IPL (Scanco, Brüttisellen, Switzerland). From these, bone volume fraction (BV/TV) and bone surface density (BS/TV) were derived. Further morphometry parameters included trabecular thickness (Tb.Th.), trabecular separation (Tb. Sp.), trabecular number (Tb. N.), connectivity density (Conn. D.) and Structure Model Index (SMI), an estimation of the plate-rod characteristic of the structure.

### μCT based finite element models (μFE)

Detailed microstructural finite element (μFE) models (element size of 10 µm) were created by a direct conversion of bone voxels to linear hexahedral elements. The number of degrees of freedom in the models ranged from 32 to 60 million.

Linear and isotropic material behaviour was assumed for bone tissue in accordance to previous studies aimed at determining the apparent Young's modulus in trabecular bone by using micro-FE analyses [22]. As the analyses were linear-elastic and we were interested in assessing differences between groups rather than absolute values of apparent elastic modulus, all elements in the μFE models were given an arbitrary tissue modulus of 15 GPa and a Poisson ratio of 0.3.

**Figure 2:**
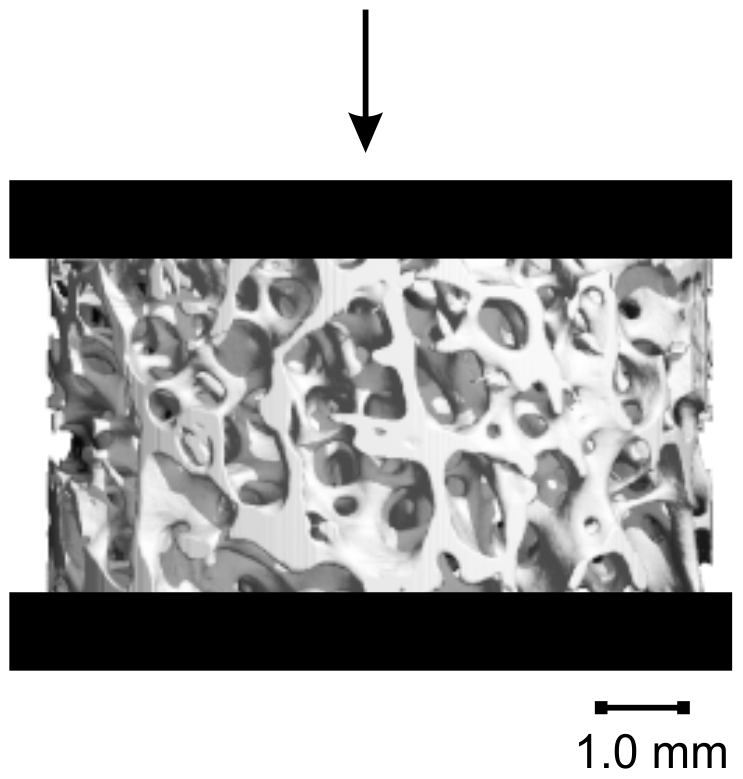
μCT-based model. μCT-based model (diam.  = 8 mm, height  = 4 mm) of bovine trabecular bone to which 1% deformation in compression is applied.

Boundary conditions that simulated the real loading situation were defined. An arbitrary displacement of 1% strain was applied to the top surface of the models without constraining the displacement in the other two directions. Only the vertical displacement of the bottom surface was constrained to simulate a friction-less contact between the specimen and the plate (Figure 2). The models were solved using ParFE, a dedicated large-scale finite element solver using 1024 cores on a Cray XT5 system [23].

The apparent elastic modulus E of each model was calculated as:
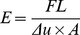
(1)where F is the calculated reaction force at the top surface while the applied displacement on the top surface (Δu), the length (L) and cross-sectional area of the sample (A), were equal to 0.04 mm, 4 mm and 50.2 mm^2^ respectively for all the models.

Effective strains corresponding to an applied load of 30 N were calculated in all elements of each sample. Effective strain, also known as “equivalent strain”, is a scalar value which summarizes the strain tensor and it has been shown to be one of the most relevant measures for predicting the bone remodeling process [24], [25]. Strain histograms (percentage of bone volume versus effective strain) for all the samples were computed.

### Statistics

A single factor analysis of variance (ANOVA) followed by a post-hoc Tukey test was performed to examine differences in label penetration, as well as morphometric and mechanical parameters between groups. Data are presented as mean ± SD and *p*<0.05 was accepted as significant.

The change in bone turnover release between the first and the second week of the experiment was analysed by means of paired t-tests (*p*<0.05 accepted as significant).

Nonparametric analyses using Spearman's rank correlation coefficient were performed to correlate the percentage of total volume experiencing high strains to the release of bone formation and resorption markers within each group of samples. For each test, a significance level of *p*<0.05 was applied. Spearman's rank correlation coefficient r_s_ were calculated to reflect the trends in the relationships. All statistical analyses of the data were performed using the statistical software package SPSS (SPSS Inc., Chicago, IL, USA).

## Results

### Bone turnover biomarkers

Both the ProCI and NTx intra-assay variation between two repeated measurements on the same sample was lower than 5%. The inter-assay variation between two measurements performed on the same sample by two different kits was lower than 8%.

**Figure 3:**
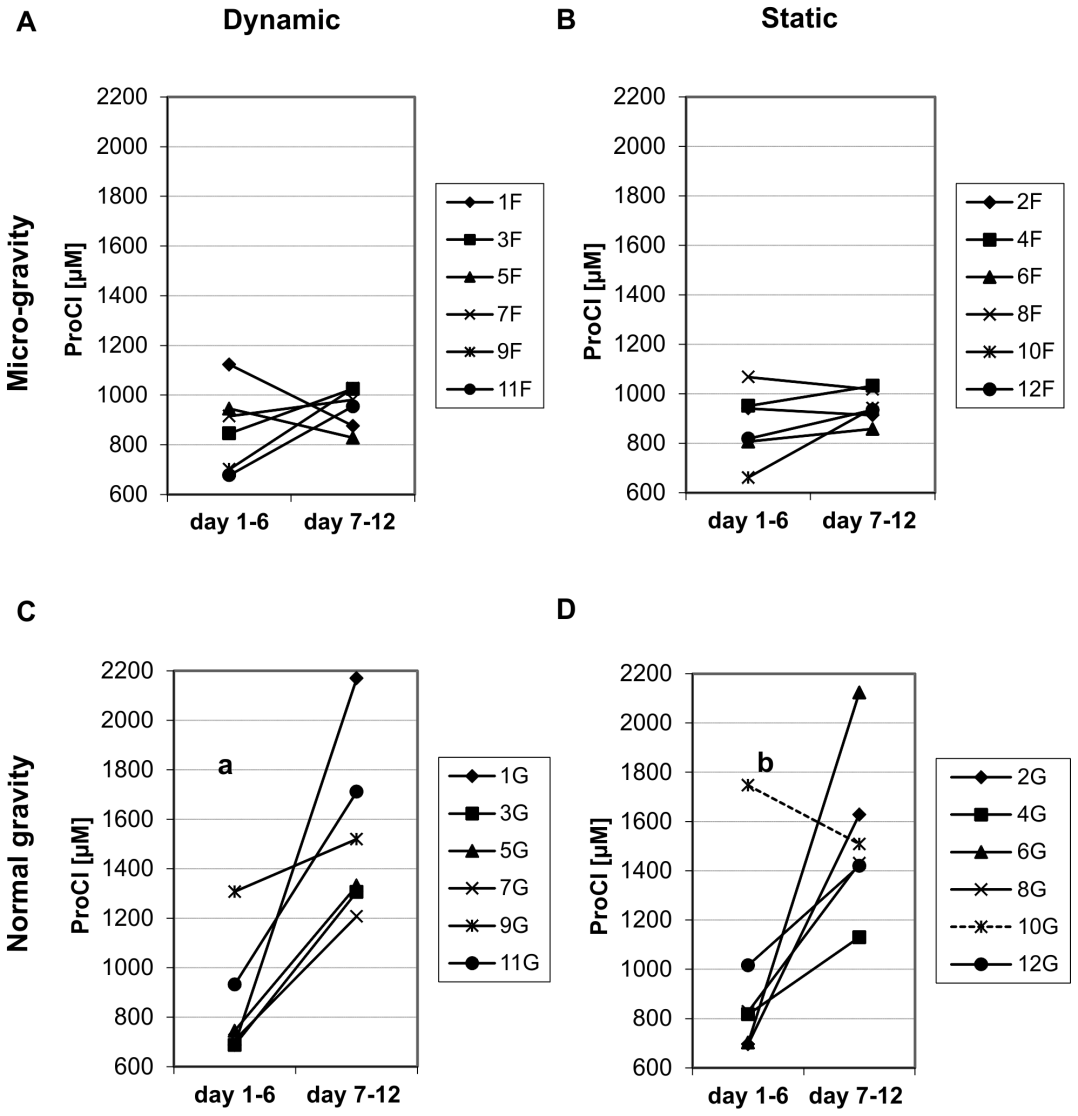
ProCI (µM) released by each bone sample. ProCI (µM) released by each sample in the culture medium during the first week (medium analyzed at the 6^th^ day) and the second week (medium analyzed at the 12^th^ day) of the experiment for the four groups of samples. A) Data relative to the samples dynamically loaded at microgravity; B) Data relative to the samples statically loaded at microgravity; C) Data relative to the samples dynamically loaded at normal gravity; D) Data relative to the samples statically loaded at normal gravity. ^a^ *p*<0.05, n = 6. ^b^ *p*<0.05, n = 5 (sample ‘10G’ is an outlier and was excluded for statistical analyses).

The biomarker data of the statically loaded group at normal gravity showed the presence of one potential outlier relative to the release of bone formation marker in the first week (Figure 3 D). The specific value was 115% higher than the average of the remaining values. The interquartile range rule confirmed this to be an outlier; hence, this value (“10G”) was removed from all further analyses in this study.

**Figure 4:**
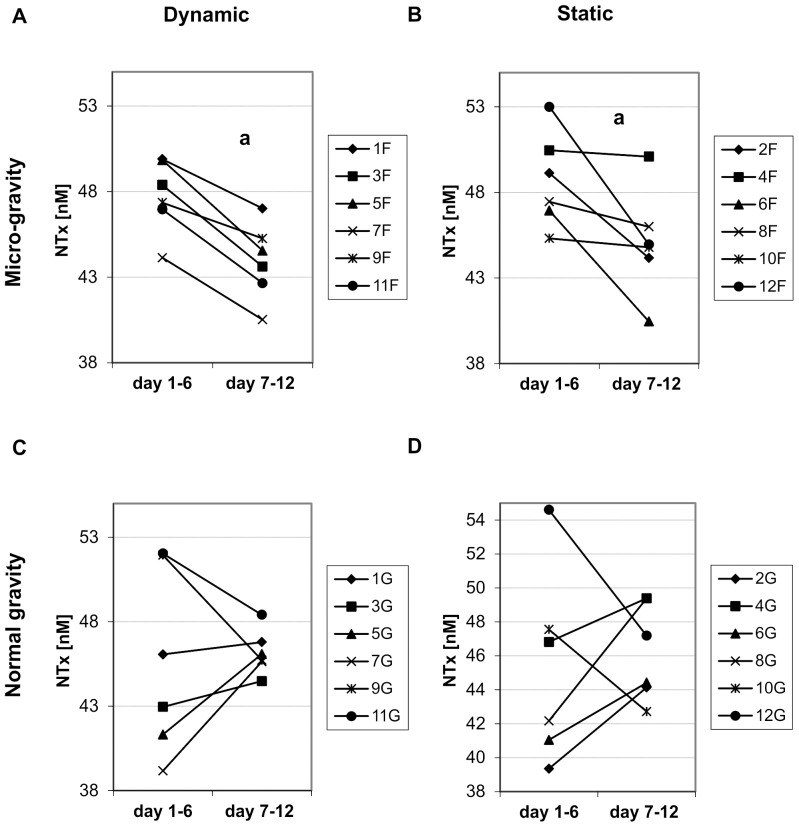
NTx (nM BCE) released by each bone sample. NTx (nM BCE) released by each sample in the culture medium during the first week (medium analyzed at the 6^th^ day) and the second week (medium analyzed at the 12^th^ day) of the experiment for the four groups of samples. A) Data relative to the samples dynamically loaded at microgravity; B) Data relative to the samples statically loaded at microgravity; C) Data relative to the samples dynamically loaded at normal gravity; D) Data relative to the samples statically loaded at normal gravity. ^a^ *p*<0.05, n = 6.

The bone formation marker ProCI showed an increased release from the first to the second week of the experiment in response to static and dynamic loading at normal gravity (by 90.4% and 82.5% in average, respectively) (Figure 3). The change in ProCI release at microgravity was not significant (Figure 3). The bone resorption data (Figure 4) indicated a decrease in NTx release in the second week from the explants cultured at microgravity in response to static and dynamic loading; on average, the decrease was 7.5% and 8.0% for the static and dynamic loading group, respectively; the change in NTx release from explants cultured at normal gravity was not significant.

### Bone histology

**Figure 5:**
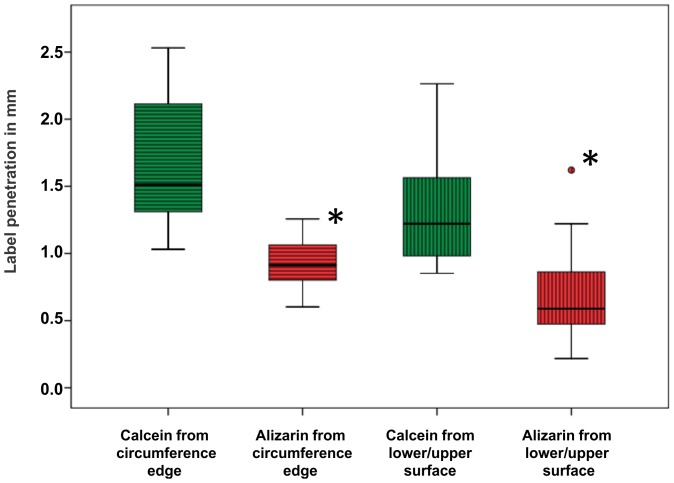
Label penetration depth at two penetration sites (from lower and upper surfaces, and from circumference edge). Calcein green penetration, which can be defined as time point zero, was significantly increased (*p<0.01) compared to the alizarin complexon penetration (end time point). Box plots show the median line, the 25% and the 75% quartiles which define the box, the 1.5x interquartile-range whiskers, as well as outliers (○).

The comparison of label penetration of the single labels (calcein green or alizarin complexon) at either penetration site (upper and lower surface, or circumference edge) showed no significant differences between the experimental groups. However, the overall calcein penetration depth at either penetration site was significantly greater than alizarin penetration (p = 0.001) (Figure 5). Specifically, the penetration for calcein and alizarin from the circumference edge was 1.65 mm (+/−0.45 mm) and 0.93 mm (+/−0.18 mm), respectively; penetration of the labels from the upper and lower surfaces was lower with 1.33 mm (+/−0.39 mm) on average for calcein green and 0.69 mm (+/−0.33 mm) for alizarin complexon (p<0.0001).

**Figure 6:**
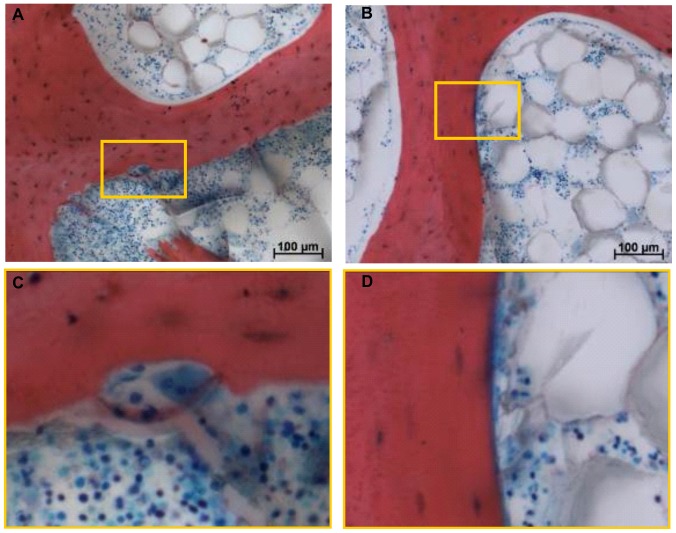
Light microscopic micrographs of Giemsa and eosin stained sections of the bone explants. A) Micrograph shows bone matrix labelled (red), haematopoietic bone marrow (blue), and the presence of a multinucleated osteoclast (blue) lying in its resorption lacunae; B) Micrograph shows bone matrix (red), haematopoietic bone marrow (blue), and the presence of an osteoid seam (blue); C) Close-up of osteoclast; D) Close-up of osteoid. Close-up scale bar represents 25 µm.

To investigate the morphological state of the cultured bone explants, Giemsa and eosin staining was performed. The presence of mainly red bone marrow was detected in the samples (Figure 6 A). Furthermore, several intact osteoclasts were detected after 20 days experimental culture time (Figure 6 A, C). Osteoclasts as multinucleated mature bone-resorbing cells have a life span of only 12.5 days *in vivo* [26]. The presence of osteoid seams - newly deposited, non-mineralised matrix – could also be visualised (Figure 6 B, D).

**Figure 7:**
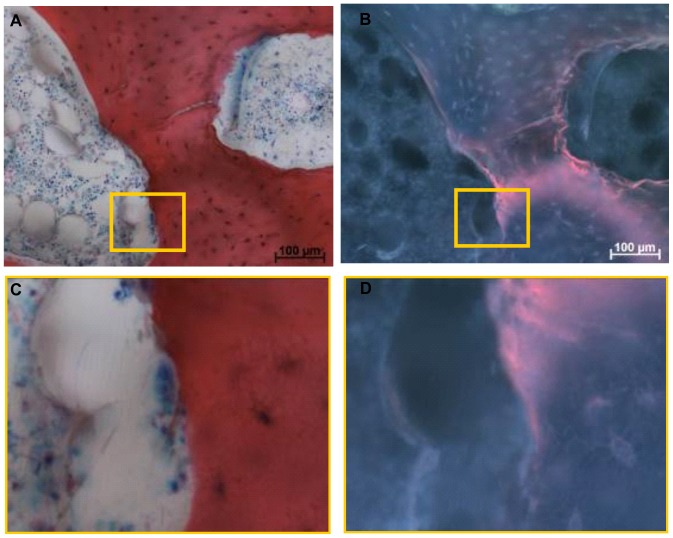
Light microscopy and fluorescence micrographs of sections of a bone explant dynamically loaded at micro-gravity. A) Light microscopic image showing an osteoclast within its resorption lacunae; B) Fluorescence micrograph showing the same area of cell activity with the corresponding alizarin bone surface labelling. C) Close-up of osteoclast as visualized by light microscopy; D) Close-up of the same area of cell activity as visualized by fluorescence labelling. Close-up scale bar represents 25 µm.

**Figure 8:**
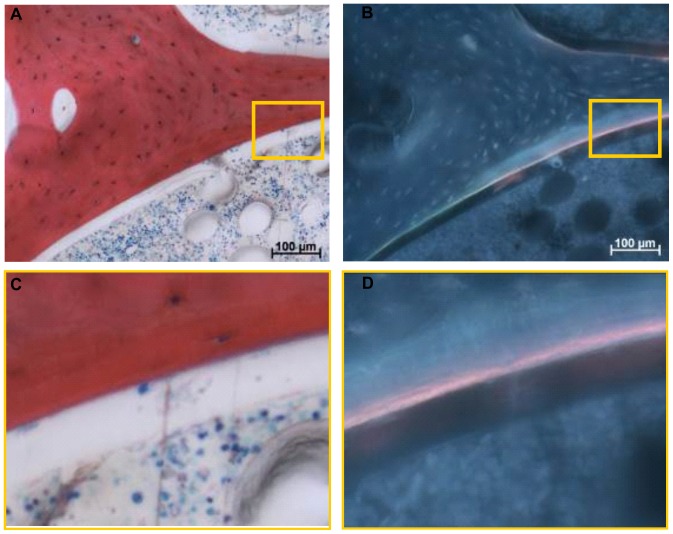
Light microscopic and fluorescence micrographs of sections of a bone explant dynamically loaded at micro-gravity. A) Light microscopic image showing an osteoid seam; B) Fluorescence micrograph showing the same area of cell activity with the corresponding alizarin bone surface labelling. C) Close-up of osteoclast as visualized by light microscopy; D) Close-up of the same area of cell activity as visualized by fluorescence labelling. Close-up scale bar represents 25 µm.

It was possible, to correlate areas of bone formation and resorption activities seen by the detection of osteoid seams and intact osteoclasts respectively with fluorescent double labelling in order to demonstrate that labelling was specific to the cellular activity during the mission. Both applied labels bind to all ‘free calcium binding sites’, therefore, labels can bind during bone formation but also during bone resorption processes when free calcium binding sites are made available by the activity of osteoclasts. Direct labelling of resorption sites can thus come about because the osteoclasts expose binding sites for the fluorochromes. Labelled resorption lacunae remain visible in the section, but only when the resorption process does not proceed further after application of the fluorochrome [27], [28]. The four micrographs in figure 7 show the overlapping osteoclast presence inside its resorption lacuna and alizarin complexon labelling, demonstrating that bone resorption can be correlated with the labelling. The presence of osteoid and overlapping alizarin complexon labelling (Figure 8) highlights the localisation of calcium label to bone forming sites.

### Bone quality

No statistically significant differences were found for any of the morphometric and mechanical properties for the four groups of sample (Table 1).

**Table 1:** Bone structure and mechanical competence for the 4 groups of samples.

	Microgravity		Normal gravity	
Variable	dynamic	static	dynamic	static
BV/TV (%)	16.50±2.80	14.58±2.48	15.61±2.44	14.83±1.76
BS/TV (1/mm)	3.02±0.31	2.83±0.29	2.92±0.20	2.82±0.33
Tb.Th. (mm)	0.15±0.014	0.14±0.007	0.15±0.01	0.15±0.012
Tb. N. (1/mm)	1.39±0.11	1.34±0.09	1.35±0.09	1.33±0.15
Tb. Sp. (mm)	0.65±0.07	0.68±0.06	0.68±0.057	0.70±0.09
Conn. D. (1/mm3)	6.07±1.31	5.33±0.48	5.59±0.58	5.21±1.06
SMI	0.85±0.25	0.94±0.30	0.85±0.25	0.97±0.20
E app (MPa)	940.60±637.95	560.90±171.50	733.13±195.50	636.27±270.53

Morphometric parameters (BV/TV  =  bone volume fraction, BS/TV  =  bone surface density, Tb.Th.  =  trabecular thickness, Tb.N.  =  trabecular number, Tb.Sp.  =  trabecular separation, Conn. D.  =  connectivity density, SMI  =  structure model index) and calculated apparent Young's modulus (E app) for the 4 groups of samples are indicated. No statistical differences were found. Values are expressed as mean ± standard deviation (n = 6 samples/group).

### Strain distribution versus bone turnover

Bone turnover in the second week was associated with the volume of highly strained bone (Table 2). In the dynamically loaded groups NTx levels were negatively correlated to the percentages bone strained above 222 (r_s_ = −1) and 373 microstrains (r_s_ = −0.812) at normal and microgravity, respectively. For the statically loaded groups no statistically significant correlations between the volume of loaded bone and NTx were found.

**Table 2:** Outcomes of the correlation analyses between the bone turnover markers (ProCI and NTx) released by the samples with the volume (expressed in percentage) strained above different strain effective levels.

		ProCI *versus* volume percentage strained above threshold			NTx *versus* volume percentage strained above threshold		
		strain threshold (μstrains)	*R^2^*	Direction	strain threshold (μstrains)	*R^2^*	Direction
**Microgravity**	**dynamic**	-	-	-	373	0.60	↓^a^
**Microgravity**	**static**	1090	0.80	↑^a^	-	-	-
**Normal gravity**	**dynamic**	191	0.45	↓^a^	222	0.94	↓^a^
**Normal gravity**	**static**	-	-	-	-	-	-

Reported are the strain thresholds for which the coefficient of determination R^2^ was maximum (only when the *p*-value associated with the Spearman correlation analyses was significant). ‘Direction’ indicates whether the bone markers were increasing (↑) or decreasing (↓) with increasing the strained bone volume.

a , *p*<0.05.

ProCI levels were decreasing with the volume of highly strained bone for the group dynamically loaded at normal gravity; the highest linearity was detected at 191 microstrains (r_s_ = −0.829). In the statically loaded group at microgravity ProCI levels were positively correlated to the percentages bone strained above 1090 microstrains (r_s_ = 0.829). No statistically significant correlations between the volume of highly strained bone and ProCI were found for the statically loaded at normal gravity and the dynamically loaded group at microgravity.

## Discussion

In this study we evaluated bone quality of the trabecular bone samples after being cultured in normal and microgravity and being subjected to either a static or a dynamic load. Statistical analyses on morphometric parameters and apparent stiffness did not reveal significant differences between the treatment groups.

Histological analysis demonstrated structural maintenance of the tissue specimens throughout the experimental procedure with the presence of red bone marrow and morphological intact osteoclasts. Fluorescent labelling with calcein green and alizarin complexon showed correlation with osteoclast and osteoblast activity; yet this was found in a few samples only (Fig. 6, 7, and 8). Label penetration analysis showed significant differences. Both labels represent experimental time points, calcein green specifies the time point zero prior to launch, while alizarin complexon specifies the end point of the experiment after explant recovery. Therefore, the decrease in label penetration of alizarin complexon in comparison to calcein green describes a reduction in label penetration over time.

The analysis of the bone turnover markers showed an increase in bone formation marker (ProCI) release from the first to the second week of the experiment at normal gravity; this suggested that both static and dynamic loading stimulated the bone formation processes in the cancellous bone samples. In addition, it appeared that mechanical stimulation was less effective in inducing bone formation at microgravity compared to normal gravity. The changes in bone resorption marker (NTx) release indicated that bone resorption is reduced at microgravity in response to both static and dynamic loading. Overall, the effect of mechanical loading appeared regulated mainly by an increase of bone formation at normal gravity and by a decrease in bone resorption at microgravity.

Previous studies have revealed that bone formation can decrease and bone resorption can increase during space flight [29] and bed rest conditions [30]. The precise bone response cannot be compared directly to our findings, as these studies referred to bone remodeling processes at microgravity *in vivo*, which were quantified by serum or urinary assays, hence, included the influence of the systemic regulations. Moreover, our study concentrated on the comparison of dynamic versus static loading and lacks a true disuse group due to space constraints on the spacecraft. Yet, our study fills a gap in the understanding of the bone adaptive responses to microgravity. Specifically, in addition to the experiments mentioned above, demonstrating decreased bone formation in humans due to microgravity, it has been demonstrated that dissected bones, i.e. without the influence of systemic signals, also experience less osteoblast activity under microgravity [31]. In addition, it has been shown that osteoblast cultures show reduced activity under microgravity with respect to normal gravidty conditions [32], [33]. The present study showed microgravity to be inhibitory towards bone cell function too as confirmed by the reduction of osteoblast activity from day 6 to day 12 of the experiment in bones subject to mechanical loading.

The third and final goal of this study was to investigate whether a strain threshold would exist above which bone response was activated. In the group dynamically loaded at normal gravity, strains of similar order of magnitude appeared to regulate bone formation and resorption processes (191 microstrains and 222 microstrains, respectively). The group dynamically loaded at microgravity showed significance for bone resorption only and for a strain threshold of 373 microstrains. These results might confirm bone adaptation to be driven by tissue strain magnitude lower than 500 microstrains applied at 30 Hz [14] and this to be valid both at normal and microgravity.

Also in the group statically loaded at microgravity, we found that a strain threshold existed at which the change in bone formation with the percentage volume was significant; this value was substantially higher (1090 microstrains) compared to the dynamically loaded groups. This implicates that bone responds to static mechanical strain and that higher strain magnitudes will be necessary when the complementary effect of loading frequency is missing. Whether this bone response may lead to a net anabolic or catabolic effect requires additional investigation, e.g. comparisons with data relative to reference (unloaded) bone samples.

Several limitations can be identified for this study. First, we used six samples per group. A proper prospective power analysis was not possible because of the lack of comparable studies. From a post-hoc power analysis of the morphometric data we determined that at least 25 samples per group had been needed in order to detect statistically significant differences with p<0.05 at a statistical power of at least 0.81. Due to limitations related to volume as well as weight and power consumption, this amount of samples would have not been realistic to include in the mission [34]. The challenges of performing bioreactor experiments under microgravity are immense as there are severe restrictions on the access to the device and limitations on its size and weight. This reduces the potential sample size to the minimum and eliminates the possibilities for repeats, hence making statistical analysis almost impossible. It also requires careful experimental design to obtain as much data as possible from a limited sample set. Nevertheless, in this study, valuable data was obtained which provides insights into the behavior of cyclically loaded samples cultured under this very challenging environment. Recent developments in *in* vivo micro-CT scanning would allow for pre-flight scanning of the samples. That could reduce the required number of samples, because each sample would act as its own control [35].

Second, we used cancellous bone taken from the bovine sternum based on the finding that sternum cancellous bone explants do respond to dynamic loading [15]. Yet, it is questionable whether this is the bone with the best adaptive response as the negative changes in BMD during weightlessness and microgravity have primarily been detected in weight-bearing bones [36] found in the lower extremities and the pelvis.

Third, although the computational analyses provided information about the local mechanical stimuli (effective strain magnitude) that would occur in the samples at day 12 of the experiment, the release of bone markers that might have been driven by such stimuli remained unknown. In this study we hypothesized that the release of bone turnover markers from day 6 – day 12 would be indicative for the bone turnover at day 12. Unfortunately, no data are available that may support this assumption.

In conclusion, we found mechanical loading to affect trabecular bone remodelling by different mechanisms at normal and microgravity. Additional studies with extended experimental time and increased samples number appear necessary for a further understanding of the anabolic potential of dynamic loading on bone quality and mechanical competence. We found low strain magnitudes to drive bone turnover when applied at high frequency, and found this to be valid at normal as well as at microgravity. Further investigations are indispensable for better understanding the linkage between mechanical stimuli and bone tissue response at normal and microgravity.
